# Third-Party Ranks Knowledge in Wild Vervet Monkeys (*Chlorocebus aethiops pygerythrus*)

**DOI:** 10.1371/journal.pone.0058562

**Published:** 2013-03-08

**Authors:** Christèle Borgeaud, Erica van de Waal, Redouan Bshary

**Affiliations:** 1 University of Neuchâtel, Institute of Biology, Neuchâtel, Switzerland; 2 University of St Andrews, School of Psychology, St Andrews, United Kingdom; 3 Inkawu Vervet Project, Mawana Game Reserve, Swart Mfolozi, KwaZulu Natal, South Africa; Université Paris 13, France

## Abstract

The Machiavellian/Social Intelligence Hypothesis proposes that a complex social environment selected for advanced cognitive abilities in vertebrates. In primates it has been proposed that sophisticated social strategies like obtaining suitable coalition partners are an important component of social intelligence. Knowing the rank relationships between group members is a basic requirement for the efficient use of coalitions and the anticipation of counter-coalitions. Experimental evidence for such knowledge currently exists in only few species. Here, we conducted rank reversal playback experiments on adult females belonging to three different groups of free-ranging vervet monkeys (*Chlorocebus aethiops pygerythrus*) to test their knowledge of the female hierarchy. Playbacks simulating rank reversals (subordinate aggressing a dominant) induced longer looking times than playbacks simulating a dominant aggressing a subordinate. Vervet monkey females therefore seem to compute the rank relationships between other females. Our results suggest that detailed social knowledge about rank relationships may be widespread in primates and potentially also in other species living in stable groups.

## Introduction

There is enormous variation in both absolute and relative vertebrate brain size [1]. The complexity of a species’ social life has been identified as one important factor promoting the evolution of large brains [2], [3], though exactly which aspects of social life require larger brains remains largely unknown. The most general hypotheses are the social brain hypothesis [3], [4] and the Machiavellian intelligence hypothesis [5]–[7], which in their generalist form include all possible aspects of social life.

An important basic requirement for sophisticated social strategies is the knowledge not only of one’s own relationships with all other group members but also of the relationships between other group members. In general, individuals of many species obtain such information through ‘eavesdropping’ on social interactions in a communication network [8]. Evidence for this capacity has been provided in a variety of vertebrate taxa [9]. However, subjects typically needed to remember few interactions of few individuals [10]–[13]. The existing evidence thus ignores the possibility that variation in brain size could at least in part reflect quantitative differences in knowledge/memory [14]. The knowledge that group living animals have about third-party relationships has been studied mainly in primates [2], [15]–[19]. Early experiments strongly suggest that primates recognise mother-offspring relationships [20], [21]. Furthermore, there is experimental evidence for more detailed social knowledge in baboons and in chimpanzees. Using playbacks simulating rank reversals Bergman et al. [17] demonstrated that female baboons know the entire female hierarchy in their group. In chimpanzees, individuals exaggerate their screams when aggressed in the presence of a third-party that is dominant over their aggressor [19], suggesting both detailed knowledge about the group’s hierarchy and strategic use of the information. Outside primates, evidence that individuals know the entire hierarchy within their group has been provided on pinyon jays [12]. Thus, while current explicitly experimental evidence in primates is restricted to two particularly large brained species [22], [23], observational evidence suggests that detailed knowledge about the relationships between other group members may well be widespread in primates [16], [18] and more generally in species living in large stable groups [24]. Nevertheless, more explicit experimental studies seem warranted. Here, we studied vervet monkeys, which belong to the guenons [25], the most diverse old world monkeys’ clade. Previous research indicates that vervets monkeys are not only able to group mother-offspring pairs and group membership of neighbours [2], [21] but also that they may already use quite sophisticated social strategies, like forming coalitions that are affected by recent grooming interactions [2], and adjusting the amount of grooming given to others in response to favours received [26].

The social structure of vervet monkeys closely resembles that of savannah baboons, with female philopatry and matrilinear ranks [2]. We therefore used an experimental design similar to the approach developed for baboons [15], [17] to test whether vervet females know the rank relationships between other females. Previous studies found that subjects looked at speakers longer in response to incongruent playbacks mimicking conflicts out-of-line with the hierarchy than those that were congruent with the existing hierarchy [15], [17]. We predicted that if vervet monkey females know all rank relationships between other females they should also look longer at speakers if ranks are reversed.

## Methods

The study was conducted from October 2011 until April 2012 at the Inkawu Vervet Project, Mawana game reserve (S 28° 00.327; E 031° 12.348), Kwazulu Natal, South Africa. Subjects were adult females from three habituated groups of vervet monkeys. Ignoring infants, the Baie Dankie group included 37 individuals (4 males, 10 females and 23 subadults and juveniles), Noha 24 individuals (2 males, 10 females and 12 subadults and juveniles) and Ankhase 25 (3 males, 6 females and 16 subadults and juveniles).

Playback experiments mimicked the methodology of a previous study on baboons [17] where a sequence of calls was played that simulated a conflict between two females. Sequences consisted of two aggression calls from the aggressor followed by one distress call from the victim. In the control sequence a dominant appears to aggress a subordinate while in the experimental sequence a subordinate appears to aggress a dominant. Calls were recorded during foraging experiments with a directional Sennheiser K6/ME 66 microphone and a Marantz PMD 660 recorder. Each call was played only once. The spacing between the two aggression calls (90 ms) as well as between the second aggression call and the distress call (40 ms) was kept constant (Audacity v. 1.3). Average length of the sequences was 2.3 seconds. The duration of distress calls was quite variable but for each subject the length was the same between the control and the experiment. The amplitude from each aggression call (65 dB) and distress call (78 dB) was normalised in Praat (v. 5.2.28) so that it was as similar as possible to a natural occurring conflict.

We used calls from all 26 females to test 16 females (six from Noha, eight from Baie Dankie and two from Ankhase). Subjects invariably ranked two positions above or below the two dyads that were used as signallers for the two playback trials. Within the four individuals used for playbacks, the highest ranking invariably aggressed a partner two ranks lower in the hierarchy, while the lowest ranking aggressed a partner two ranks higher in the hierarchy. For example, the alpha female was tested with female 3 against 5 as control and 6 against 4 on the rank reversal trial, while the 10^th^ female was tested with 5 against 7 as control and 8 against 6 on the rank reversal trial.

Each subject heard the two call sequences in trials separated by at least 24 h, with the order of control and experimental playbacks being counterbalanced between subjects. The subject and the two signallers were monitored by two or three observers who were in constant radio contact. Playbacks were only conducted when the two signallers were out of sight of the subject and at least 30 m away, Calls were played out of a Logitec S715i. Speakers were placed behind a bush or in the grass about 15 m away from the subject so that they were hidden from this one. From the subject’s perspective the speakers were placed such that the playback came from the general direction in which the donors of the playback calls currently were. Playbacks were started when the subjects were resting or foraging and looking away from the speakers. Subjects were filmed 10 s prior the playbacks until 30 s after the end of the sequence.

Frame by frame (Virtualdub v. 1.6.19) analysis was used to score the duration the subject looked towards the speakers after the onset of the second signaller’s call. Scoring stopped as soon as the individual moved its face away from the speakers’ direction. Videos were analysed by the experimenter (CB) and by a naive person (RB). Assessments matched within 2 frames (8/100 s) in 80% of videos, while 20% yielded quite differing results. It turned out that mismatches were likely if the subject had briefly looked away while the playback was still ongoing. We agreed that this should be dismissed as the complete information had not yet been available to the subject. With this in mind RB (still naïve) reanalysed the ambiguous videos to provide the values used for the statistics. The analysis was conducted using SPSS (v. 20).

### Ethical Note

The study was approved by the relevant local authority, Ezemvelo KZN Wildlife and by the University of Cape Town, South Africa. Our setup involved only playbacks of conflict vocalizations and video recordings. Playbacks were used only up to twice a day on different subjects, to avoid increase of stress or conflicts.

## Results

**Figure 1:**
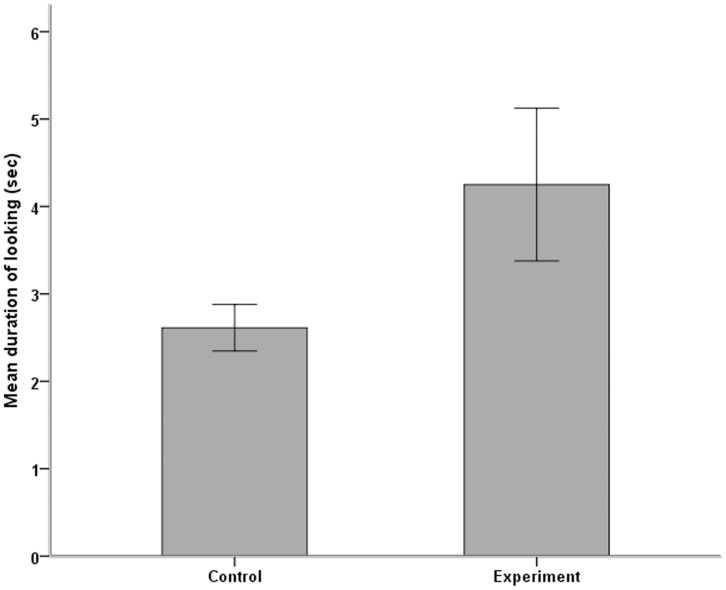
Duration (mean and SE) that individuals looked towards the speakers during control (dominant aggresses subordinate) and experimental (subordinate aggresses dominant) playbacks.

Individuals looked significantly longer towards the speakers during incongruent experimental sequences than during congruent control sequences (Mean time looking congruent = 2.612 (SE = 0.266), incongruent = 4.25 (SE = 0.872), N = 16, Wilcoxon Signed rank test: Z = −2.068, p = 0.039; figure 1). There was no correlation between the rank of the individual distressing and the time spent looking nor a correlation between the rank of the subject and the time spent looking (Spearman tests: N = 16, both p>0.2).

## Discussion

Our results demonstrate that female vervet monkeys distinguish between playbacks that suggest a female conflict congruent with the hierarchy and playbacks that suggest a rank reversal. Thus we could replicate the main result found in previous studies on baboons [15], [17]. The data suggest that similar evidence for baboons and chimpanzees can be generalized at least for old world monkeys and apes and potentially to species living in stable groups. Nevertheless, with respect to the quantitative arguments we have put forward in the introduction, we note that vervet monkeys live in smaller groups than either baboons or chimpanzees and hence our subjects needed to process less information than individuals in the latter two species. It would hence be interesting for the future to find an experimental design that explicitly tests whether individuals of different species have a maximal number of third-party relationships they can track that correlates with the species typical group size. Such experiments would bring us closer to actual testing of the social brain hypothesis, which proposes that brain size imposes a constraint on maximal group size due to an individual’s limitation in the ability to track third-party relationships beyond that [3], [27].

### Methodological Considerations and Perspectives

A potential criticism of the experimental design could be that subjects respond stronger to distress calls from more dominant individuals because they occur less frequently, and that this simple rule, rather than detailed knowledge about the hierarchy, may have caused the significant results. We consider this alternative explanation unlikely to be correct for two reasons. First, we did not find positive correlations between the apparently distressing individual’s rank and the duration subjects looked at the speaker. Second, the three subjects that looked longer during control situations were exposed to distress calls from high ranking females during the experimental situation. According to the alternative hypothesis, these trials should have evoked particularly long attention spans but they did not. Similarly, Cheney et al. [15] controlled for the novelty of a sequence by showing that adding a third aggressive call from a female dominant to both (that could post hoc explain the previous sequence of calls) indeed yielded low attention by subjects. We also note that the rank of subjects was apparently not important for our results as there was no correlation between the rank of the subject and the time spent looking, and the three subjects that looked longer during the control situations did not occupy extremely high or low ranks. Thus, it appears that female vervet monkeys indeed know the entire female hierarchy of their group.

Our study did not fully replicate the experiments by Bergman et al. [17] on baboons as we did not have a third group of playbacks that involved rank reversals within matrilines. We could not replicate these data on the vervet monkeys because in contrast to the study on baboons we did not have detailed information about the pedigree of subjects. Bergman et al. [17] did not find any significant differences between subjects’ responses to ‘correct’ rank interactions and reversals within matrilines. While they interpreted this result as evidence for a nested representation of the hierarchy, Penn et al. [28] argued that the very same data yield evidence for an absence of such a nested representation. In any case the non significant result seems difficult to interpret as it could also mean that baboons do not track rank relationships within matrilines. Thus, it appears that the third treatment group would not have yielded results that allow conclusions beyond knowledge about third-party rank relationships.

As a final methodological remark, we note that due to the lack of information about the pedigree of subjects we cannot exclude that subjects sometimes reacted more strongly because one of the individuals involved in the playbacks was a full or half sister. Also, the genetic relationships between the two individuals used for playbacks will have been variable. However, such uncontrolled effects should increase the variance in the data and hence favour the null hypothesis that vervet monkeys do not know the relative ranks of group members. Hence our approach was conservative, making the significant result robust. Furthermore an advantage of our study compared to Bergman et al. [17] is that we kept the difference in rank between individuals used for playbacks constant.

In the future, it would be interesting to test the females’ knowledge about the males’ hierarchy and vice versa, as well as investigating the juveniles’ knowledge about third-party relationships. Most importantly, we can now start to expand on earlier research [2] and study how vervet females use their detailed knowledge on relative ranks for strategic Machiavellian-like behaviour. Having stable coalition partners appears to be important for reproductive success [29] but it remains unclear how important social competence [30], [31] is in comparison to kinship to achieve a high fitness.
